# Supplementary material for: Needs- and user-oriented development of contactless camera-based telemonitoring in heart disease–Results of an acceptance survey from the Home-based Healthcare Project (feasibility project)
Source: PLoS One. 2023 Mar 7;18(3):e0282527. doi: 10.1371/journal.pone.0282527 (PMC9990940; doi:10.1371/journal.pone.0282527)
Supplement: S1 Fig — (PDF) [file pone.0282527.s001.pdf]

*S1 Fig. Questionnaire for patient survey (UTAUT determinants inserted in blue) - **German***

**Erwarteter Nutzen**	stimmt völlig	stimmt	stimmt eher	stimmt eher nicht	stimmt nicht	stimmt überhaupt nicht	**Bemerkungen/Anmerkungen/ Nachfragen Wurde Frage nicht verstanden?**
Diese neue Messtechnik fände ich nützlich für die Betreuung meiner chronischen Erkrankung.	O	O	O	O	O	O	
Durch die Nutzung dieser neuen Messtechnik, würden meine Chancen steigen, eine gesundheitliche Gefahrensituation schneller zu erkennen und frühzeitiger Hilfe zu bekommen.	O	O	O	O	O	O	
Durch die Nutzung dieser neuen Messtechnik bräuchte ich weniger Zeit für die selbstständige Messung meiner Vitalwerte.	O	O	O	O	O	O	

**Erwarteter Aufwand**	stimmt völlig	stimmt	stimmt eher	stimmt eher nicht	stimmt nicht	stimmt überhaupt nicht	
Ich fände es einfach, diese neue Messtechnik anzuwenden.	O	O	O	O	O	O	
Ich würde keine Schwierigkeiten haben, den Umgang mit der neuen Messtechnik zu erlernen.	O	O	O	O	O	O	

**Sozialer Einfluss**	stimmt völlig	stimmt	stimmt eher	stimmt eher nicht	Stimmt nicht	stimmt überhaupt nicht	
Meine Freunde und Familienangehörigen würden der Ansicht sein, dass ich diese neue Messtechnik benutzen sollte.	O	O	O	O	O	O	
Mein Hausarzt würde der Ansicht sein, dass ich diese neue Messtechnik benutzen sollte.	O	O	O	O	O	O	

Einstellung gegenüber der Technologie	stimmt völlig	stimmt	stimmt eher	stimmt eher nicht	stimmt nicht	stimmt überhaupt nicht	
Ich würde dieser neuen Messtechnik vertrauen, dass sie korrekt arbeitet.	O	O	O	O	O	O	
Die Nutzung dieser neuen Messtechnik…							
… wäre eine gute Idee.	O	O	O	O	O	O	
… würde Spaß machen.	O	O	O	O	O	O	
… fände ich interessant.	O	O	O	O	O	O	

Beeinflussung durch die Technologie	stimmt völlig	stimmt	stimmt eher	stimmt eher nicht	stimmt nicht	stimmt überhaupt nicht	
Die Nutzung dieser neuen Messtechnik würde meine Arzt-Patienten-Beziehung beeinflussen.	O	O	O	O	O	O	
Die Nutzung dieser neuen Messtechnik würde meinen bisherigen täglichen Rhythmus/Routine beeinflussen.	O	O	O	O	O	O	

Integrierbarkeit in den Tagesrhythmus	stimmt völlig	stimmt	stimmt eher	stimmt eher nicht	stimmt nicht	stimmt überhaupt nicht	
Diese neue Messtechnik könnte ich gut in meinen Tagesrhythmus integrieren.	O	O	O	O	O	O	

Beabsichtigte Nutzung	stimmt völlig	stimmt	stimmt eher	stimmt eher nicht	stimmt nicht	stimmt überhaupt nicht	
Wenn mir diese neue Messtechnik zu Hause zur Verfügung stehen würde, würde ich sie benutzen.	O	O	O	O	O	O	

Selbstwirksamkeit	stimmt völlig	stimmt	stimmt eher	stimmt eher nicht	stimmt nicht	stimmt überhaupt nicht	
Ich würde mir eine eigenständige Nutzung der neuen Messtechnik zutrauen, unter der Bedingung,…							
… dass ich einen telefonischen Ansprechpartner hätte.	O	O	O	O	O	O	
… dass die Messtechnik eine integrierte Hilfe-Funktion anbieten würde.	O	O	O	O	O	O	

Ängstlichkeit	stimmt völlig	stimmt	stimmt eher	Stimmt eher nicht	stimmt nicht	stimmt überhaupt nicht	
Ich hätte Bedenken, diese neue Messtechnik zu nutzen.	O	O	O	O	O	O	
Ich hätte Angst, dass ich bei der Nutzung der neuen Messtechnik Fehler machen könnte.	O	O	O	O	O	O	
Die ständige Messung, immer wenn ich vor der Kamera stehe, würde mich verunsichern.	O	O	O	O	O	O	
Ich würde an der Qualität und Zuverlässigkeit der neuen Messtechnik zweifeln.	O	O	O	O	O	O	

**Fragen zur Person**
Geschlecht:<br>☐ weiblich<br>☐ männlich
Alter:<br>☐ 18-29 ☐ 60-69<br>☐ 30-39 ☐ 70-79<br>☐ 40-49 ☐ 80-89<br>☐ 50-59 ☐ 90-99
Wie schätzen Sie Ihre Fähigkeiten im Umgang mit neuen Technologien im Allgemeinen ein (Handy, Smartphone, Tablet, Computer, …)?<br>☐ **hoch** (ich komme mit den meisten Technologien sehr gut zurecht)<br>☐ **mittelmäßig** (ich komme mit den meisten Technologien gut zurecht) ☐<br>**gering** (ich komme mit den meisten Technologien schwer zurecht)

Perspektivisch wäre ein Einsatz dieser neuen Messtechnik in der Telemedizin denkbar.

Das heißt, dass die **mit Hilfe der Kamera gemessenen Körperfunktionswerte** auch **über räumliche Entfernung hinweg** zu Ihrem Hausarzt **übermittelt** werden.

Dazu notwendig sind:

- eine Kamera in die Sie eine Weile schauen
- eine Internetverbindung zur Übertragung der Daten

1. Wo sollte sich die Kamera bei Ihnen zu Hause befinden? (Handy, Computer, Spiegel, Fernseher, welches Zimmer)

2. Sollte die Kamera immer an sein? Oder nur, wenn Sie messen möchten?

3. Möchten Sie immer eine Rückmeldung zu Ihren gemessenen Werten haben? Wenn ja, wie sollte diese erfolgen (visuell, akustisch)?

4. Sollten die gemessenen Werte regelmäßig an Ihren behandelnden Arzt weitergeleitet werden?

5. Worin würden Sie den Nutzen einer solchen Technik sehen?

   Für Sie selbst?

   Für Ihren Hausarzt?

6. Hätten Sie Bedenken bei der Anwendung einer solchen Technik?
   Wenn ja, welche?

7. Haben Sie einen Internetanschluss (Kabel oder Handy)?
   Falls nein:

   - Würden Sie sich selbstständig einen Internetanschluss zulegen, um die neue Messtechnik nutzen zu können?

   - Würden Sie sich einen Internetanschluss zulegen, wenn Sie bei der Einrichtung und Wartung von einem Techniker unterstützt würden?

   - Wären Sie bereit, die Kosten für den Internetanschluss selbst zu tragen?

8. Sonstige Anmerkungen

Vielen Dank für Ihre Beteiligung!

S1 Fig. Questionnaire for patient survey (UTAUT determinants inserted in blue) – ***Translation in English - Survey conducted in German***

Performance expectancy	strongly agree	agree	rather agree	rather disagree	disagree	strongly disagree	**Remarks/enquiries**<br>**Was the question not understood?**
I would find this new measuring technique useful for the care of my chronic illness.	O	O	O	O	O	O	
By using this new measurement technology, my chances of recognizing a dangerous health situation more quickly and getting help earlier would increase.	O	O	O	O	O	O	
By using this new measurement technology, I would need less time to measure my vital signs myself.	O	O	O	O	O	O	

Effort expectancy	strongly agree	agree	rather agree	rather disagree	disagree	strongly disagree	
I would find it easy to use this new measurement technique.	O	O	O	O	O	O	
I would have no trouble learning to use the new measurement technology.	O	O	O	O	O	O	

Social influence	strongly agree	agree	rather agree	rather disagree	disagree	strongly disagree	
My friends and family would think that I should use this new measurement technique.	O	O	O	O	O	O	
My GP would think I should use this new measurement technique.	O	O	O	O	O	O	

Attitude toward using technology	strongly agree	agree	rather agree	rather disagree	disagree	strongly disagree	
I would trust this new measurement technology to work correctly.	O	O	O	O	O	O	
The use of this new measurement technique…							
... would be a good idea.	O	O	O	O	O	O	
... would be fun.	O	O	O	O	O	O	
... would be interesting.	O	O	O	O	O	O	

Influence of the technology	strongly agree	agree	rather agree	rather disagree	disagree	strongly disagree	
The use of this new measurement technology would affect patient relationship.	O	O	O	O	O	O	
The use of this new measuring technology would influence my previous daily rhythm/routine.	O	O	O	O	O	O	

Integrability into the daily routine	strongly agree	agree	rather agree	rather disagree	disagree	strongly disagree	
I could easily integrate this new measuring technique into my daily routine.	O	O	O	O	O	O	

Behavioral intention	strongly agree	agree	rather agree	rather disagree	disagree	strongly disagree	
If this new measurement technology were available to me at home, I would use it.	O	O	O	O	O	O	

Self-efficacy	strongly agree	agree	rather agree	rather disagree	disagree	strongly disagree	
I would trust myself to use the new measurement technology independently, provided that...							
… that I would have a telephone contact person.	O	O	O	O	O	O	
... that the measurement technology would offer an integrated help function.	O	O	O	O	O	O	

Anxiety	strongly agree	agree	rather agree	rather disagree	disagree	strongly disagree	
I would have reservations about using this new measurement technique.	O	O	O	O	O	O	
I would be afraid that I could make mistakes when using the new measurement technology.	O	O	O	O	O	O	
The constant measurement whenever I stand in front of the camera would unsettle me.	O	O	O	O	O	O	
I would doubt the quality and reliability of the new measurement technology.	O	O	O	O	O	O	

Sociodemographic issues
Gender:<br>☐ female<br>☐ male
Age:<br>☐ 18-29 ☐ 60-69<br>☐ 30-39 ☐ 70-79<br>☐ 40-49 ☐ 80-89<br>☐ 50-59 ☐ 90-99
How do you rate your skills in dealing with new technologies in general (mobile phone, smartphone, tablet, computer, ...)?<br>☐ **high** (I'm very comfortable with most technologies)<br>☐ **moderate** (I'm comfortable with most technologies)<br>☐ **low** (I have difficulty using most technologies)

A use of this new measuring technology in telemedicine would be conceivable. This means that the body function values measured with the help of the camera are also transmitted to your family doctor over a distance. This requires:

- a camera that you look at for a while
- an internet connection to transfer the data

1. Where should the camera be located in your home? (mobile phone, computer, mirror, TV, which room)

2. Should the camera always be on? Or only if you want to measure?

3. Would you always like to have feedback on your measured values? If so, how should this be done (visually, acoustically)?

4. Should the measured values be regularly forwarded to your doctor?

5. Where would you see the benefit of such a technique?

   For herself?

   For your GP?

6. Would you have any concerns about using such a technique?

   If yes, which?

7. Do you have an internet connection (cable or mobile)?

If not:

- Would you set up an Internet connection yourself to use the new to use measurement technology?

- Would you consider getting an internet connection if you are at the setup and maintenance supported by a technician?

- Would you be prepared to bear the costs for the Internet connection yourself?

8. Other Notes

Thank you for your participation!
