# Supplementary material for: Needs- and user-oriented development of contactless camera-based telemonitoring in heart disease–Results of an acceptance survey from the Home-based Healthcare Project (feasibility project)
Source: PLoS One. 2023 Mar 7;18(3):e0282527. doi: 10.1371/journal.pone.0282527 (PMC9990940; doi:10.1371/journal.pone.0282527)
Supplement: S1 Table — (PDF) [file pone.0282527.s002.pdf]

S1 Table. Categorization of acceptance (regarding the intended use).

	**Acceptance high**	**Acceptance moderate**	**Acceptance low**
**Intended use**	completely agree + agree	mostly agree + mostly disagree	disagree + completely disagree
