# Supplementary material for: Needs- and user-oriented development of contactless camera-based telemonitoring in heart disease–Results of an acceptance survey from the Home-based Healthcare Project (feasibility project)
Source: PLoS One. 2023 Mar 7;18(3):e0282527. doi: 10.1371/journal.pone.0282527 (PMC9990940; doi:10.1371/journal.pone.0282527)
Supplement: S2 Table — (PDF) [file pone.0282527.s003.pdf]

S2 Table. Categorization of the determinants.

Number of items	Determinant	High	Moderate	Low
3	**Performance expectancy**	completely agree + agree	mostly agree + mostly disagree	disagree + completely disagree
2	**Effort expectancy***	disagree + completely disagree	mostly agree + mostly disagree	completely agree + agree
2	**Self-efficacy**	completely agree + agree	mostly agree + mostly disagree	disagree + completely disagree
2	**Social influence**	completely agree + agree	mostly agree + mostly disagree	disagree + completely disagree
4	**Anxiety**	completely agree + agree	mostly agree + mostly disagree	disagree + completely disagree
1	**Integratability**	completely agree + agree	mostly agree + mostly disagree	disagree + completely disagree
2	**Influence of the technology**	completely agree + agree	mostly agree + mostly disagree	disagree + completely disagree
		**Positive**	**Neutral**	**Negative**
4	**Attitude toward technology**	completely agree + agree	mostly agree + mostly disagree	disagree + completely disagree

* The items concerning effort expectancy are phrased as polar opposites, hence a high level of agreement corresponds with low effort.
