# Supplementary material for: Needs- and user-oriented development of contactless camera-based telemonitoring in heart disease–Results of an acceptance survey from the Home-based Healthcare Project (feasibility project)
Source: PLoS One. 2023 Mar 7;18(3):e0282527. doi: 10.1371/journal.pone.0282527 (PMC9990940; doi:10.1371/journal.pone.0282527)
Supplement: S3 Table — (PDF) [file pone.0282527.s004.pdf]

S3 Table. Example of categorization for determinants with multiple items.

**Performance expectancy**	completely agree	agree	mostly agree	mostly disagree	disagree	completely disagree
**Assignment of value**	**6**	**5**	**4**	**3**	**2**	**1**
I find this new measuring technology useful for the monitoring of my chronic disease.	O	**x**	O	O	O	O
By using this new measuring technology, my chances of more quickly recognizing a medically risky situation and receiving timely help would increase.	O	O	**x**	O	O	O
By using this new measuring technology, I would need less time to measure my vital parameters.	**x**	O	O	O	O	O
	**Mean value = 5 (performance expectancy = high**)					
