# Supplementary material for: Needs- and user-oriented development of contactless camera-based telemonitoring in heart disease–Results of an acceptance survey from the Home-based Healthcare Project (feasibility project)
Source: PLoS One. 2023 Mar 7;18(3):e0282527. doi: 10.1371/journal.pone.0282527 (PMC9990940; doi:10.1371/journal.pone.0282527)
Supplement: S4 Table — (DOCX) [file pone.0282527.s005.docx]

*S4 Table: Inclusion and exclusion criteria for study participation*

| Inclusion criteria | Exclusion criteria |
| --- | --- |
| Age ≥ 18 years | Presence of the diagnosis: moderate to severe dementia |
| Presence of at least one of the following diagnoses:   - chronic heart failure (at least NYHA stage 2) - coronary artery disease - hypertensive heart disease - aortic valve stenosis (at least moderate) - chronic obstructive pulmonary disease (COPD, at least gold stage 3) | Existence of an abnormal score in the geriatric basic assessments: Mini-Mental-State-Examination (< 20 points), clock test (0-5 points) or Dem-Tect-Test (≤ 8 points) |
| Sufficient ability to provide information (i.e. to be able to answer simple questions about one's own state of health/well-being) | Presence of a quantitative disturbance of consciousness (drowsiness, somnolence, sopor, coma) |
| Sufficient command of the German language (i.e. to be able to answer the survey questions) | Known substance abuse (alcohol or drugs) |
| Understanding of the patient information and consent to the study possible | Severely limited ability to provide information |
