# Supplementary material for: Needs- and user-oriented development of contactless camera-based telemonitoring in heart disease–Results of an acceptance survey from the Home-based Healthcare Project (feasibility project)
Source: PLoS One. 2023 Mar 7;18(3):e0282527. doi: 10.1371/journal.pone.0282527 (PMC9990940; doi:10.1371/journal.pone.0282527)
Supplement: Attachment — Submitted filename: Rebuttal letter.doc [file pone.0282527.s007.doc]

**PLOS ONE**

**Submission of research article**

Needs- and user-oriented development of contactless camera-based telemonitoring in heart disease – Results of an acceptance survey from the Home-based Healthcare Project (Feasibility Project)

Peggy Borchers, MPH

Research assistant

Telefon:	0351 458 89228

Telefax:	0351 458 888 3561

E-Mail:	Peggy.Borchers@ukdd.de

Dresden, June 30, 2022

Dear Sir or Madam,

This manuscript was submitted to PLOS Digital Health in May 2022 and rejected for the following reason:

*„Specifically: The submitted manuscript covers an interesting topic of contactless telemonitoring for heart diseases. The authors have evaluated a contactless-camera telemonitoring unit using a small group of participants. I reviewed this manuscript in detail and I feel there are some vital issues including sample size and the methodology. The sample size is low for us to generalise the findings to a larger audience and the methodology section can be further strengthened with use of statistical correlation tools.  If possible, a qualitative evaluation with a mixed methods can add immense value to this project. Kindly also review the PLOS Digital Health publication criteria for better understanding of our scope.” (Padmanesan Narasimhan, MBBS MPH PhD, Section Editor PLOS Digital Health)*

Small sample size:

The sample size is small because it is a feasibility study. This is also addressed in the Limitations section. We have emphasized this in the manuscript to avoid any confusion about the study design (yellow in the manuscript).

Methodology:

We did not use interference statistical methods because of the small sample size.

Mixed methods:

Unfortunately, this is no longer possible afterwards.

Due to the design of the feasibility study, we think that the manuscript fits the scopes of *PLOS ONE* better. We hope you will consider publishing our study in *PLOS ONE* and look forward to your reply.

Best regards, on behalf of all authors

Peggy Borchers, MPH
