# Supplementary material for: Needs- and user-oriented development of contactless camera-based telemonitoring in heart disease–Results of an acceptance survey from the Home-based Healthcare Project (feasibility project)
Source: PLoS One. 2023 Mar 7;18(3):e0282527. doi: 10.1371/journal.pone.0282527 (PMC9990940; doi:10.1371/journal.pone.0282527)
Supplement: Attachment — Submitted filename: Response to Reviewers_.doc [file pone.0282527.s008.doc]

**PLOS ONE**

**Submission of research article**

Needs- and user-oriented development of contactless camera-based telemonitoring in heart disease – Results of an acceptance survey from the Home-based Healthcare Project (Feasibility Project)

Peggy Borchers, MPH

Research assistant

Telefon:	0351 458 89228

Telefax:	0351 458 888 3561

E-Mail:	Peggy.Borchers@ukdd.de

Dresden, January 09, 2023

**Dear Reviewers,**

Thank you very much for the time you have taken for the review and your constructive comments. Here are the points mentioned, which we went into in detail.

**Dear Reviewer 1**,

Thank you very much for your intensive examination of the present article and the constructive suggestions.

Reviewer #1: The paper explores user acceptance of contactless camera-based telemonitoring in heart disease patients, as a part of The Home-based Healthcare Project. I found it interesting to read, well written (although language fluency should be improved) and coherent.

The small sample size is not a problem in my opinion: it is a feasibility study and the "Limitations" section properly addresses the issue. Inference statistical methods cannot be used due to the small sample size: the authors understand that and give themselves self-limitations in the scope of the paper.

Before publication, I suggest a few minor revisions:

* In the Development of the Questionnaire" section, I would not write that "According to which, the acceptance of a technology is expressed in the use of that technology". The original UTAUT paper by Venkatesh is already centered on the intention to use.

🡪 Has been reworded (line 143-144)

* Reference 18 (Venkatesh et al., 2003) should be complemented by more recent work by Venkatesh, expanding the Unified Theory (see, for example, Venkatesh, V., J. Thong, and X. Xu. 2016. “Unified Theory of Acceptance and Use of Technology: A Synthesis and the Road Ahead.” Journal of the Association for Information Systems 17 (5): 328–376)

🡪 Reference has been included.

* The evaluation measurements taken during the study should be listed in the text. Blood pressure is mentioned somewhere, but I would like to read the full catalogue.

🡪 Measured parameters and devices used were listed. (line 103 – 105)

* I suggest a thorough language revision. Sentences like "The measures to boost acceptance paid special attention this" in the abstract need adjustments.

🡪 since only one reviewer wants a linguistic revision, this point is requested from the editor

🡪 The sentence has been removed.

**Dear Reviewer 2,**

Thank you very much for your intensive examination of the present article and the constructive suggestions. We picked them up for the most part and, in our opinion, this has contributed to the improvement of the article.

Reviewer #2: Dear authors,

Thank you for reviewing your article. The paper covers an interesting topic but is missing relevant information like new literature and a discussion reflecting the overall limitations due to low sample size. The low sample size is fine for feasibility study but the conclusion/results are presented in a way that it seems to be absolute. Presenting conclusion resulting of 3 patients (group 2) should be in a more descriptive/qualitative manner. Further I give my comments for specific sections:

Introduction:

Actual literature is missing (ESC guidelines, TIM-HF2, OSICAT, German decision about reimbursement). Especially telemedicine in Heart Failure is a very fast developing research topic and has provided a lot of new research content within the last year. As the authors are from Germany they should reflect the decision of the GEmeinsamen Bundesausschuss to pay for telemedicine in HF as part of standard care (see decision process starting in December 2020 until Qualitätssicherungsvereinbarung in March 2022). The introduction also not differentiate between non-invasive and invasive telemonitoring; at least in one sentence this differentation should be addressed.

- Thank you for these hints. We incorporated them. (line 34-41)

In the further paper Heart failure is not mentioned any more – are the survey respondents HF patients?

- Heart failure was one of the inclusion criteria, along with other heart diseases. Added to the text. (line 116-121)

Please also provide a definition of “elderly”.

🡪 The corresponding age from the cited publication was added. (>70 years)

Methods

As this feasibility analysis was part of a overall study – has the overall study a registration number (e.g. DRKS)?

- no, the overall study was not registered and has no registration number by DRKS, since it was not a RCT

Line 85: Why written informed consent is in Brackets. Written informed consent is crucial for any study (see Good Clinical Practice). Also a statement to be in line with declaration on Helsinki is missing.

🡪Brackets has been removed. Accordance with the Declaration of Helsinki has been added. (line 94-95)

Why only 19 patient were recruited, when 20 were planned. The authors names “pragmatic reasons” for case low case number. Did they looking for patients regarding specific inclusion and exclusion criteria? Please specify, as this could be big bias.

- Reasons for the 19 study patients and the corresponding inclusion and exclusion criteria were added. (line 113-121 + S4)

The UTAUT is only valid in the English and only for the complete version. Please discuss a possible limitation due to translation and reduction of questions in the limitation sections.

- A possible limitation due to this has been included. (line 363-365)

Line 97: Are results of the study available.

- No, the evaluation of this data is still ongoing and is being carried out by the Institute for Biomedical Engineering at the Technical University of Dresden.

Which 250 measurements were performed?

- The evaluation measurements described in lines 101-105.

Section data collection

The first sentence is not necessary. Why the survey ends (December 2020) before the study (July 2021)? Please specify

- The first sentence has been deleted.
- Because then all study participants were interviewed after several evaluation measurements had been carried out on the study patients and they were familiar with the measurement technology (line 174-176)

Line 158/159: As the case number is very low in the results section it is better to present absolute instead of relative numbers.

- Sorry, I cannot understand the objection, since both (absolute + relative) are always specified in the results section.

As group 2 included only 3 persons the results are not comparable. This need to be discussed.

- The small group (n=3) was explicitly mentioned again in the limitations. (line 354)

Why 3 groups were grouped when only 2 were presented? What are the (mean) values to be categorized in each group? Is the group “moderate/low” a mix of this 2 groups? According the results in Table 2 nobody was categorized as “low acceptance”. Why was the column then named in this way?

- Thank you for this hint. It was formulated more clearly that Group 2 included people with low and moderate acceptance (line 218-220)
- In Table 2 it has been made clearer what the mean value belongs to. The calculation of the mean is described in the Analyses chapter.

Results

Why only 18 patients participated in the evaluation survey?

- All study patients were invited to the survey interviews. But one study patient didn’t want to take part in the interview. Has been supplemented. (line 204-206)

How long did the patients use the measuring system when they answered the questionnaire.

- As described in lines 127-130, the patients did not use the measuring system themselves. “*The acceptance survey was already oriented toward the next step in the prospective development of this measuring technology into a TMA for independent use by patients. This involves a planned TMA undergoing development based on the results of the evaluation study.”*
- However, it was added in the text that an average of 5 evaluation measurements were carried out before the interview. (line 205-206)

Why the questionnaire was not used after the overall study was finished?

- Exactly for the reason mentioned in the previous point. This acceptance survey was carried out in order to incorporate the results into the development of the TMA (which did not exist at this time).

Patients idea: visual and acoustic combined – why is this the same group as acoustic? Were Multiple answers possible?

🡪 Yes, multiple answers were possible. Has been supplemented. (line 254)

What means “followed by the study” (line 252)?

- Study in this case it means something like workroom. To be on the safe side, we added room. (line 263)

In the results section results of both groups are often presented together. I would suggest to do this for all results and not to provide to groups. Difference in the groups could be discussed qualitative; but due to the n=18 vs. n=3 patients in groups these results are not comparable. For this reason figure 2 cannot be so conclusive; please name it in a way like “possible characteristics”. When a sample only consists of 3 person, all results can be random.

- It was added to the caption figure 2 that these are determined characteristics of the study patients surveyed, with n=3 (line 243)
- Added an additional note in Limitations (line 354)

Line 236: How sun burn can be happen due to use of the camera?

- Right, using the camera can not cause sun burn. The presence of sun burn can affect the measurement results. Now it has been explained in more detail in the text. (line 273 )

Line 252: “This involved mostly women…” As 17% of 18 participant are only 3 persons, 2 persons have to be female. A conclusion in this way is not correct. The discussion should present absolute numbers to reflect this bias of a low case number.

- was converted to an absolute number (line 288-289)

TMA structure for HF patients (personal, technical) is actually clearly defined by the G-BA; reimbursement is possible in Germany (decision in March 2021, reimbursement numbers defined in January 2022). The decision was not done as the survey was performed but it should be discussed as e.g. a fear of overwhelming the primary care physician is not longer necessary. Physician can decided whether to perform TMA itself or by a telemedical centre.

- Thanks for the hint. That is correct. However, the results section is simply about the concerns that the patients have expressed. But we have included an addition in the discussion. (line 305-309)

Line 281: What means modern technology?

- was added in brackets (e.g. smartphone, tablet, computer) (line 323)

Also, the authors could discuss that acceptance does not mean compliance or adherence. A long term analysis is needed for this.

- Thank you for this hint. Additions were made to the text. (line 378-379)

Best regards, on behalf of all authors

Peggy Borchers, MPH
