# Supplementary material for: Needs- and user-oriented development of contactless camera-based telemonitoring in heart disease–Results of an acceptance survey from the Home-based Healthcare Project (feasibility project)
Source: PLoS One. 2023 Mar 7;18(3):e0282527. doi: 10.1371/journal.pone.0282527 (PMC9990940; doi:10.1371/journal.pone.0282527)
Supplement: Attachment — Submitted filename: Response to Reviewers.docx [file pone.0282527.s009.docx]

**PLOS ONE**

**Submission of research article**

Needs- and user-oriented development of contactless camera-based telemonitoring in heart disease – Results of an acceptance survey from the Home-based Healthcare Project (Feasibility Project)

Peggy Borchers, MPH

Research assistant

Telefon:	0351 458 89228

Telefax:	0351 458 888 3561

E-Mail:	Peggy.Borchers@ukdd.de

Dresden, February 15, 2023

Dear Editor,

Thank you very much for considering our manuscript for publication. We are happy to revise this according to the comments.

- revise the manuscript addressing comments from Reviewer 2;

🡪 Has been done.

- include in the manuscript (or in the supporting information) items utilized for measurement of all determinants (in English);

🡪 S1 Fig has also been translated into English (now S1 Fig_rev).

- copy edit the manuscript correcting possible errors, for instance a the end of Table 1 eliminate “Error! Reference source not found”.

🡪 Has been removed.

**Dear reviewer 1,**

Thank you very much for the time you have taken for the review again. We are very pleased that you are satisfied with the revision.

**Dear reviewer 2,**

Thank you very much for the time you have taken for the review again. Here are the points mentioned, which we went into in detail.

Dear authors, thank you for taking note of my recommendations. I still do not understand the evaluation concept completly.

- To explain: as described in lines 120-123, the acceptance survey was aimed at the next step in the further development (after the evaluation measurements).

You are writing that 250 evaluation measurements were taken by the study nurses in the patients house (methods sections). In your review comments you mentioned that „an average of 5 evaluation measurements were carried out before the interview.“ (otherwise 250 measurement divided by 19 patients does not average 5 measurements).

- We hope that the additional explanations (row 167-174) and addition (107-108) make this easier to understand.

Are these measurements taken by the patients themself?

- No, they were taken by the study nurses as described in lines 106-108.

Also you write, that the evaluation was taken by phone. Why was the survey not carried out directly after the measurements by the study nurse? How long was the time interval between measurement and evaluation survey?

- The survey was originally intended to be carried out at the end of the evaluation study. Due to the onset of the corona pandemic, it was already carried out by telephone in the course of the course. Comments on this were added to lines 167-174.
- Average evaluation measurements per study patient were added to rows 107-108.

Best regards, on behalf of all authors

Peggy Borchers, MPH
